# Influence of Gelation Temperature on Structural, Thermal, and Mechanical Properties of Monolithic Silica Gels with Mono- and Bimodal Pore Structure

**DOI:** 10.3390/gels11030196

**Published:** 2025-03-12

**Authors:** Kai Müller, Christian Scherdel, Stephan Vidi, Gudrun Reichenauer, Moritz Boxheimer, Frank Dehn, Dirk Enke

**Affiliations:** 1 Institute of Chemical Technology, Leipzig University, 04103 Leipzig, Germany; 2 Center for Applied Energy Research, 97074 Würzburg, Germany; 3 Institute of Concrete Structures and Building Materials (IMB), Karlsruhe Institute of Technology, 76131 Karlsruhe, Germany

**Keywords:** silica gels, gelation temperature, bimodal pore structure, gas pressure-dependent thermal conductivity, Young’s modulus

## Abstract

This study explores the impact of pore volume distribution on the structural, thermal, and mechanical properties of spinodal phase-separated silica gels synthesized with poly(ethylene oxide) as a phase-separating agent. By systematically varying gelation temperatures between 20 and 60 °C, we investigate how reaction kinetics influence the resulting pore architecture, thermal conductivity, and elasticity. Nitrogen sorption, mercury intrusion porosimetry, and SEM analysis reveal a transformation from a bimodal pore structure at low temperatures, featuring interconnected macropores, to a predominantly mesoporous network with loss of bimodality. This shift in the diameter of the macropores significantly impacts the thermal insulation properties of the gels as thermal conductivity decreases from 68 to 27 mW (m·K)^−1^ due to reduced macroporosity, enhanced mesoporosity, and the Knudsen effect. Mechanical testing revealed a substantial decline in Young’s modulus with increasing gelation temperature. These changes are attributed to the interplay of mesoscale structural differences and density variations, driven by increasing gelation temperatures. While higher temperatures lead to reduced strut thickness and the loss of interconnected macropores, the substantial decline in Young’s modulus highlights the critical role of mesoscale structural integrity in maintaining mechanical stability. The findings underscore the importance of an optimized pore volume distribution in tailoring pore structure and performance characteristics, providing a pathway for optimizing silica gels for applications in thermal insulation, filtration, and catalysis.

## 1. Introduction

Silica gels are a highly versatile class of porous materials with wide-ranging applications, including thermal insulation, filtration, catalysis, and as matrices for drug delivery [1,2,3,4,5,6,7]. In thermal insulation, silica-based aerogels have gained significant attention due to their exceptionally low thermal conductivity. However, their mechanical fragility and structural instability often pose challenges for large-scale applications, particularly in environments requiring mechanical durability [8]. Additionally, silica gels play a crucial role in chromatographic separation techniques, particularly in size exclusion chromatography (SEC) like gel permeation chromatography (GPC) [9]. These methods rely on precisely controlled pore structures to separate molecules based on their hydrodynamic size, making the tunability of silica gels highly relevant for optimizing chromatographic performance [9,10]. A prominent example of silica-based chromatographic materials is the Chromolith^®^ column series by Merck KGaA (Darmstadt, Germany), which utilizes monolithic silica structures with hierarchical porosity to enhance separation efficiency and reduce backpressure [11]. Their porous structure, which can be tuned by varying the synthesis conditions, is decisive for their physical properties, such as density, thermal conductivity, and mechanical strength. The unique combination of low density, high specific surface area, and tailorable mono- or multimodal pore structure makes silica gels particularly suitable for advanced material applications [12].

In conventional sol–gel processes, silica gels are typically mesoporous, meaning their pore sizes range between 2 and 50 nm, according to IUPAC definitions [13]. However, introducing a phase separation via the addition of poly(ethylene oxide) (PEO) enables the formation of macropores, which are defined as pores larger than 50 nm [13]. Phase separation can occur through two distinct mechanisms. Binodal decomposition results in particulate structures, whereas spinodal decomposition forms a continuous, interconnected, reticulated pore network, crucial for maintaining mechanical integrity in monolithic gels [14]. The pathway of phase separation is determined by system composition and temperature [14]. In the studied system, macropore formation occurs via spinodal decomposition, as thermodynamic instability leads to the spontaneous separation of phases without nucleation, forming a co-continuous network according to the Flory–Huggins equation [15]. This process is driven by the formation of hydrogen bonds between PEO and the gelling silica phase. The formed silica–polymer complex exhibits repulsive interactions with the solvent as it grows. The system splits macroscopically into a polymer-rich silica phase and a solvent-rich phase [14]. This method allows for the creation of bimodal pore structures, where macropores coexist with mesopores, significantly altering the material’s properties. The macropores provide an opportunity to precisely modify diffusion processes within the gel, for instance, modifications according to the Thiele modulus, allowing optimization for specific applications [16,17]. The thick mesoporous struts between the macropores further stabilize the otherwise fragile silica network.

Phase separation and gelation are closely interlinked, as gelation freezes the phase-separated domains, locking in the macropore and mesopore structures. The rate of gelation, influenced by factors such as pH and temperature, thus plays a decisive role in defining the final pore architecture. An “early freezing” of the gel, driven by faster gelation kinetics, results in a finer structure, while “late freezing” allows for extended phase separation, yielding a coarser, hierarchical network with larger macropores [14,17]. For instance, the addition of acids has been shown to selectively accelerate the hydrolysis rate, while bases have been used to enhance condensation [18]. These adjustments have profound effects on the mesopore structure, allowing for precise tuning of pore sizes and distributions. In the case of gels with a bimodal pore structure formed via polymer-induced phase separation in the sol–gel process, various methods have been employed to manipulate the pore architecture. To reduce the diameter of macropores formed during phase separation, previous work has focused on adjusting the polymer content [19]. However, gels with macropore diameters below 1 µm frequently exhibit pronounced inhomogeneities, resulting in non-uniform structures that compromise material performance [20].

The role of the gelation temperature on pore structure was highlighted in previous studies, particularly by Nakanishi et al. and Meinusch et al. [17,21]. However, systematic and comprehensive investigations into the extent to which variations in gelation temperature affect the gelation process, pore formation, and the resulting thermal or mechanical properties are still lacking. This gap in understanding necessitates a deeper exploration of how temperature modulates phase separation and gelation kinetics, ultimately modifying the performance characteristics of the resulting porous materials.

This study investigates the impact of gelation temperature on the structural, thermal, and mechanical properties of silica gels synthesized with PEO as a phase-separating agent. By systematically varying the gelation temperature from 20 to 60 °C, we aim to elucidate the underlying mechanisms that govern changes in pore architecture and density and how these changes affect the thermal conductivity and mechanical stiffness of the gels. Mechanical stiffness, often quantified by Young’s modulus (E), describes a material’s resistance to elastic deformation under applied stress. In porous materials such as silica gels, E is strongly influenced by both the solid network structure and the connectivity of the pores [22], making it a crucial parameter for assessing structural integrity and mechanical performance. Special attention is given to the transition in pore morphology and its implications on the material’s insulation efficiency and structural stability, aspects crucial for applications requiring thermal insulation materials with low thermal conductivity and high mechanical resilience.

## 2. Results and Discussion

**Figure 1:**
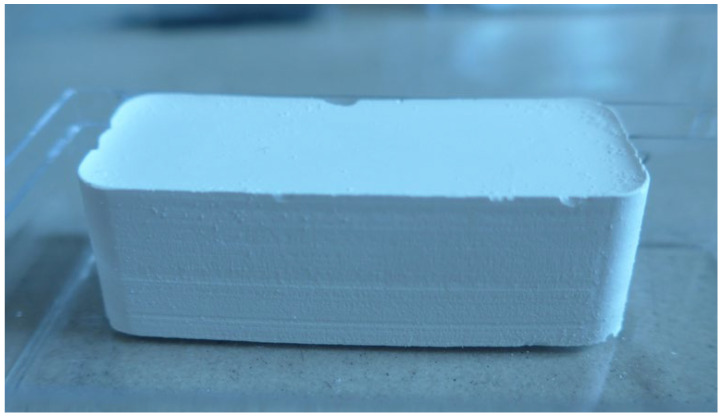
Photograph of a silica monolith (Sample G_40) with the dimensions 2 × 2 × 6 cm^3^.

To clearly identify the effect of gelation temperature on the pore structure of the silica monoliths, the composition was kept constant across all samples. Additionally, the dimensions of the monoliths were standardized at roughly 2 × 2 × 6 cm^3^ to eliminate potential scaling effects. A representative silica monolith is shown in Figure 1.

A characteristic feature of silica monoliths synthesized via the Nakanishi method is their bimodal pore structure, which consists of distinctly separated mesoporous and macroporous systems [14]. The macropore network is composed of struts with diameters in the range of several hundred nanometers. These macropore struts, in turn, are composed of a mesoporous network.

Before discussing the temperature-dependent effects, it is important to briefly address the choice of the molar ratio used in this study. The selected molar ratio was chosen based on extensive prior research in the field of phase-separating silica gels. Numerous studies, including those by Nakanishi and others, have systematically explored variations in composition and their effects on pore architecture. The chosen composition allows for an effective structural transformation with minimal experimental complexity, making it well suited for investigating the specific impact of gelation temperature on the resulting pore morphology and material properties. Given that this study aims to establish the direct relationship between gelation temperature and structural characteristics—particularly for applications in thermal insulation—the molar ratio was kept constant to isolate the temperature-dependent effects. The precise molar ratios used in this work were determined based on literature findings and preliminary optimization studies, ensuring reproducibility and consistency across all temperature variations [14].

**Figure 2:**
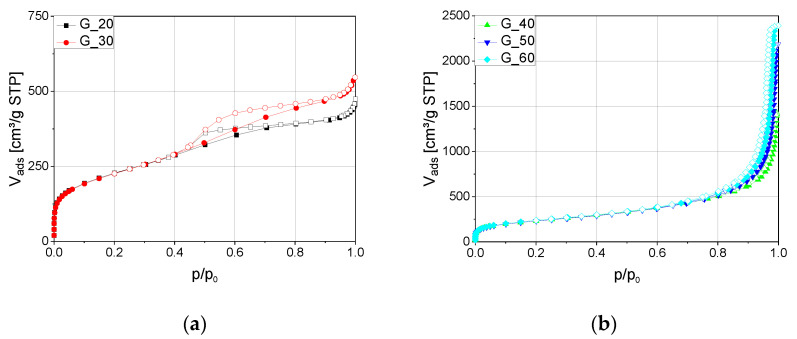
N_2_-sorption isotherms for silica gels produced at different gelation temperatures: (**a**) gels with a gelation temperature of 20 and 30 °C; (**b**) gels prepared at 40, 50, and 60 °C. Filled symbols represent the adsorption branch, while open symbols indicate the desorption branch.

Nitrogen sorption analysis was used to provide detailed information on the mesopore volume evolution as a function of gelation temperature. The adsorption–desorption isotherms are shown in Figure 2. As gelation temperature increases, the mesopore volume rises substantially, from 0.64 cm^3^∙g^−1^ at 20 °C to 3.71 cm^3^∙g^−1^ at 60 °C (Table 1), and increases its share in the total volume from 21% to 86%, i.e., the macropore volume is simultaneously reduced. With increasing gelation temperature (Figure 2b), the distinct pore domains of the mesoporous and macroporous phases gradually converge, leading to a steep increase in the adsorbed gas volume at relative pressures close to *p*/*p*_0_ = 1. This transition results in the transformation from a bimodal to a more monomodal pore system, as the disparity between mesopores and macropores diminishes.

The samples gelled at lower temperatures of 20 and 30 °C exhibit smaller mesopores due to slower condensation and extended phase separation, allowing for a more developed structure. This behavior is consistent with previous studies on phase separation in silica gels, which have shown that lower gelation temperatures promote prolonged spinodal decomposition, enabling the formation of larger phase-separated domains before gelation sets in [17,21]. In contrast, at higher gelation temperatures, the condensation reaction accelerates, and phase separation is suppressed due to the increased solubility of components. This leads to an earlier freezing of the spinodal decomposition process, limiting the development of larger macropores and resulting in a pore network dominated by smaller macropores and mesopores. The effect of gelation temperature on pore formation has been extensively studied, with research by Nakanishi et al. demonstrating that increased gelation temperatures significantly influence the size and distribution of macropores due to faster kinetics restricting phase separation [17].

**Figure 3:**
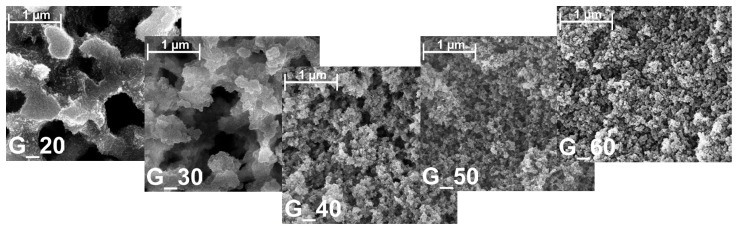
SEM images with a magnitude of 25,000 for silica gels with increasing gelation temperature from left to right.

These structural transitions were evidenced by Scanning Electron Microscopy (SEM) images, which clearly depict the morphological changes in the pore network with increasing gelation temperature (Figure 3 and Figure S1). At lower temperatures, specifically 20 and 30 °C, the SEM images display a network of large, interconnected macropores, with relatively thick silica struts that provide structural support. This interconnected macroporous structure is indicative of slower phase separation, which permits the development of larger pores and a more hierarchical network.

As the gelation temperature rises to 40 °C, the SEM images begin to show a transition in pore morphology, with the macroporous structure becoming less distinct. At this temperature, the surface appears rougher, and the silica struts become thinner, indicating a trend towards a more refined pore network. This transition continues in samples prepared at 50 and 60 °C, where the SEM images reveal a homogeneously textured surface with minimal evidence of macropores. Instead, a mesoporous structure dominates, indicating that faster gelation restricts the formation of large macropores, resulting in a mesoporous network. These structural changes suggest that higher gelation temperatures effectively “freeze” the network at an early stage, leading to a more monomodal pore structure dominated by smaller mesopores. This tunability in pore architecture is particularly relevant for applications beyond thermal insulation. For example, in SEC, precisely controlled pore structures are essential for optimizing separation efficiency, making the observed transition between macroporous and mesoporous networks highly interesting for such applications.

**Figure 4:**
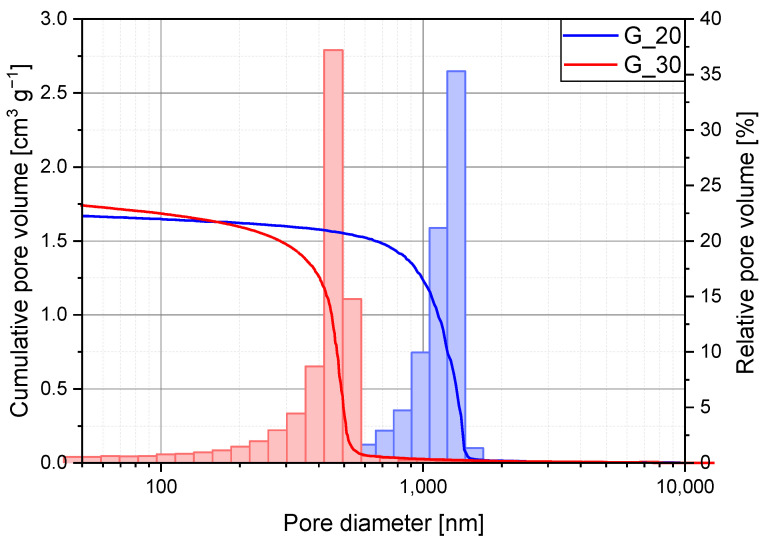
Mercury intrusion porosimetry data for the samples G_20 (blue) and G_30 (red) for a pore range between 50 and 10,000 nanometers. The cumulative pore volume is represented by the intrusion curve, while the distribution of the relative pore volume is shown by the bars.

Mercury intrusion porosimetry (MIP) data quantitatively confirm the reduction in macropore diameter with increasing gelation temperature. While MIP provides valuable insights into macroporosity, it is not well suited for detecting or characterizing mesopores in these samples, as the measurement is constrained by a maximum pressure of 400 MPa. According to the Washburn equation, this corresponds to a lower detection limit of approximately 3.7 nm. Consequently, MIP is used primarily for analyzing macropores. However, this method proved effective only for samples gelled at 20 and 30 °C, as shown in Figure 4, where a distinct bimodal pore distribution is evident. The macropores in these samples exhibit median diameters of 1149 nm and 459 nm, respectively, indicating a well-defined bimodal structure where large macropores coexist with mesopores detected via nitrogen sorption. This bimodal porosity is particularly advantageous for drying, as the presence of macropores reduces capillary forces, thereby minimizing shrinkage and preserving structural integrity. Larger macropores facilitate solvent removal, reducing capillary stress that could otherwise lead to significant shrinkage during drying [23]. At gelation temperatures above 30 °C, the pore structure becomes increasingly fragile, making MIP less reliable due to the risk of pore collapse under high pressure. To further investigate pore size distribution in samples gelled at elevated temperatures, gas pressure-dependent thermal conductivity measurements were conducted.

**Figure 5:**
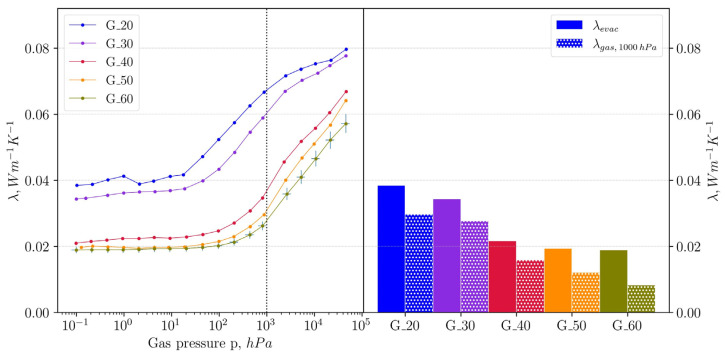
Left: measured gas pressure-dependent thermal conductivity of the samples, dotted line representing a gas pressure of 1000 hPa. Right: evacuated thermal conductivity and gas contribution to the thermal conductivity at 1000 hPa.

Gas pressure-dependent thermal conductivity measurements provide an indirect pore size estimation through variations in thermal conductivity at different gas pressures (Figure 5). Following the method developed by Vidi et al. [24], these measurements confirm the trend of decreasing macropore size with increasing gelation temperature even for samples gelled at higher gelation temperatures. Thus, sample G_20 initially shows a macropore diameter of 1212 nm, which agrees well with the results from MIP. As the gelation temperature increases, the macropore diameter initially falls to 632 nm at 30 °C and then drops drastically to 157 nm at 40 °C. A further decrease in the diameter can be observed in Table 2. Even at high gelation temperatures, residual macroporosity persists, albeit with significantly reduced diameters.

**Figure 6:**
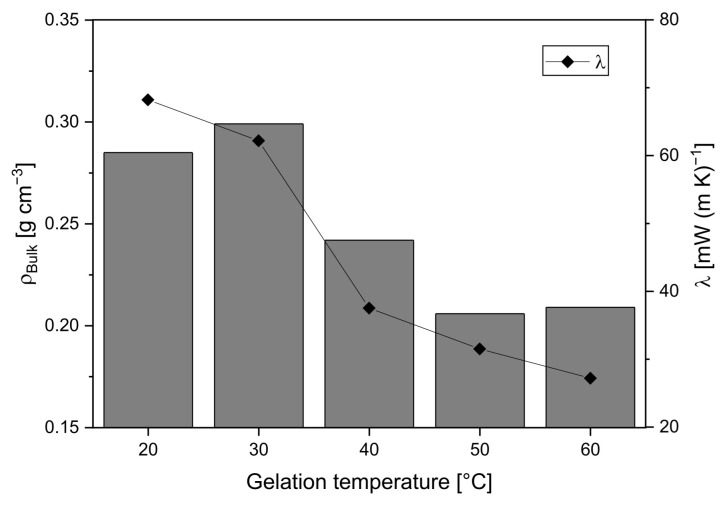
Relationship between gelation temperature and total thermal conductivity at 1 bar (dots), considering the density (bars).

The thermal conductivity results further highlight the influence of gelation temperature on the properties of these silica gels. The atmospheric thermal conductivity λ_1000hPa_ decreases from 68 mW (m·K)^−1^ for samples synthesized at 20 °C to 27 mW (m·K)^−1^ at 60 °C (Figure 6). This decrease is partly due to the reduction in bulk density, which correlates with a decline in the solid fraction available for the solid-phase heat transfer. The differences in bulk density can be attributed to variations in shrinkage behavior during the aging of the samples. It is worth noting that as the gelation temperature increased, the shrinkage of the samples decreased. This is likely due to the differences in the structure of the mesoporous network.

As the density decreases, the solid-phase thermal conductivity λ_evac_ diminishes, dropping from 38 mW (m·K)^−1^ for G_20 to 19 mW (m·K)^−1^ for G_60. A lower density implies fewer solid-phase pathways for phonon transport, which in turn reduces thermal conductivity. This trend is consistent with studies on aerogels by Lu et al., who reported that reduced density in low-temperature gels leads to lower λ_evac_ [25].

**Figure 7:**
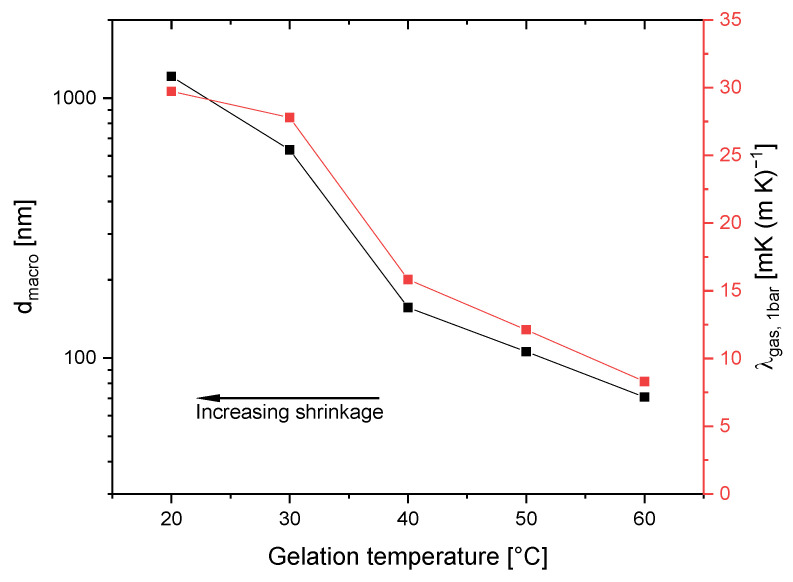
Relationship between gas-phase thermal conductivity and macropore size. The arrow indicates the increase in density.

However, the observed decline in thermal conductivity cannot be attributed solely to density. The gas-phase thermal conductivity λ_gas,1000hPa_ also shows a pronounced reduction with increasing gelation temperature, which reflects changes in the pore size of macropores and share of mesoporosity. This relationship can be viewed in Figure 7. For samples gelled at 20 and 30 °C, λ_gas,1000hPa_ is relatively high at 30 and 28 mW (m·K)^−1^, respectively, due to the predominance of larger macropores that permit conventional gaseous thermal conduction. As gelation temperature increases from 30 to 40 °C, there is a substantial drop in λ_gas, 1000hPa_, with values declining from 28 to 16 mW (m·K)^−1^. In this temperature range, the characteristic of the sample changes from predominantly macroporous to a relatively balanced ratio of mesopore volume to macropore volume of 26% to 64%.

Since the sample shows such a strong increase in the content of mesopores due to the increase in gelation temperature by 10 °C, the decline in λ_gas,1000hPa_ can partly be attributed to the Knudsen effect, where gaseous thermal conduction becomes successively limited as pore sizes approach the mean free path of gas molecules (~70 nm). In this regime, gas molecules primarily interact with pore walls, resulting in reduced thermal transport through the gas phase. The Knudsen effect becomes even more pronounced in samples prepared at higher temperatures, as the continued shrinkage of macropores and a larger fraction of mesopores result in more pores falling below the critical threshold for unrestricted gas-phase transport. That is why between 50 and 60 °C, λ_gas, 1000hPa_ continues to decline at a constant rate even though the increase in the share of mesoporosity from 78% to 86% is less pronounced. As the Knudsen number (Kn), defined as the ratio of the mean free path at a given pressure to the characteristic pore diameter, increases, gaseous thermal transport becomes more and more restricted, further lowering λ_gas,1000hPa_. This phenomenon was documented by Reichenauer, who noted that gas-phase thermal conductivity in materials is mainly dictated by the Knudsen number [26].

The substantial decrease in λ_1000hPa_ at higher gelation temperatures is therefore primarily driven by the transition from a bimodal pore structure to a predominantly mesoporous network. This transition, observed in large silica monoliths that have not been studied in such dimensions, highlights the critical role of pore architecture in disrupting heat transfer pathways. The reduction in macroporosity and the corresponding increase in mesoporosity during this transition significantly contribute to the observed decline in λ_1000hPa_. While additional factors, such as reduced density, further amplify this trend, the structural reorganization of the pore system is the dominant mechanism.

The mechanical properties of the silica gels, characterized by their E, were determined using both ultrasonic run-time measurements and compression tests. E quantifies a material’s resistance to elastic deformation under applied stress and is defined as the ratio of stress to strain in the linear elastic regime. In porous materials, E is influenced not only by the intrinsic properties of the solid silica network but also by the overall pore structure, connectivity, and density [22]. A higher porosity generally leads to a decrease in mechanical stiffness, as the solid fraction available for load-bearing is reduced [8]. This makes E a crucial parameter for applications where mechanical stability is essential, such as thermal insulation materials, chromatographic monoliths, and filtration systems, where the balance between porosity and structural integrity determines long-term performance.

Significant differences were observed between the absolute values of E obtained using the two methods. For example, the values of E for the G_20 sample were measured at 204 MPa using ultrasound, while compression tests yielded a substantially lower value of 82 MPa (Table 3). This discrepancy is likely due to the differing experimental approaches of the methods, as ultrasonic run-time measurements determine the dynamic E, while compression tests measure the static E. Moreover, the ultrasonic run-time measurements do not account for macroscopic defects or heterogeneities.

Despite these differences in absolute values, both methods exhibit the same general trend: a substantial reduction in E with increasing gelation temperature. Furthermore, the relative differences between specific samples show strong similarities across the methods. For instance, the G_30 sample, with a slightly higher density of 0.299 g∙cm^−3^ compared to G_20 at 0.285 g∙cm^−3^, demonstrates a lower E (156 MPa and 75 MPa for ultrasonic run-time measurements and compression tests, respectively). This result is contrary to the expectation that E correlates positively with density. Typically, in porous materials, an increase in density is associated with a higher fraction of load-bearing solid material, which enhances the stiffness of the structure and leads to a higher Young’s modulus [27]. This deviation can be attributed to mesoscale structural differences, where the increasing fraction of mesopores and the thinning of struts reduce the mechanical stability of the gel network. The loss of interconnected macropores and the transition to a more fragile mesoporous structure likely contribute to the weakening of the material, despite the minor variations in bulk density. Furthermore, this effect can also explain the larger difference observed in ultrasonic measurements compared to compression tests. Ultrasonic Young’s modulus is highly sensitive to the continuity and connectivity of the silica skeleton, meaning that as struts become thinner and less interconnected, wave propagation is significantly impaired, leading to a pronounced drop in E [27]. In contrast, compression tests measure the macroscopic mechanical response, which is less affected by subtle changes in strut thickness.

A different phenomenon is observed when comparing the G_50 and G_60 samples. Despite their nearly identical densities (0.206 g∙cm^−3^ and 0.209 g∙cm^−3^, respectively), a strong decrease in E—almost 40%—is observed with a 10 °C increase in gelation temperature, irrespective of the measurement method. This sharp decline suggests that mesoscale structural differences between the samples, induced by the different gelation temperatures, may play a critical role in determining the mechanical properties. Previous studies have shown that as gelation temperature increases, the accelerated condensation reaction leads to thinner silica struts and reduced network connectivity, which weakens the mechanical stability of the material despite minor changes in bulk density [17].

**Figure 8:**
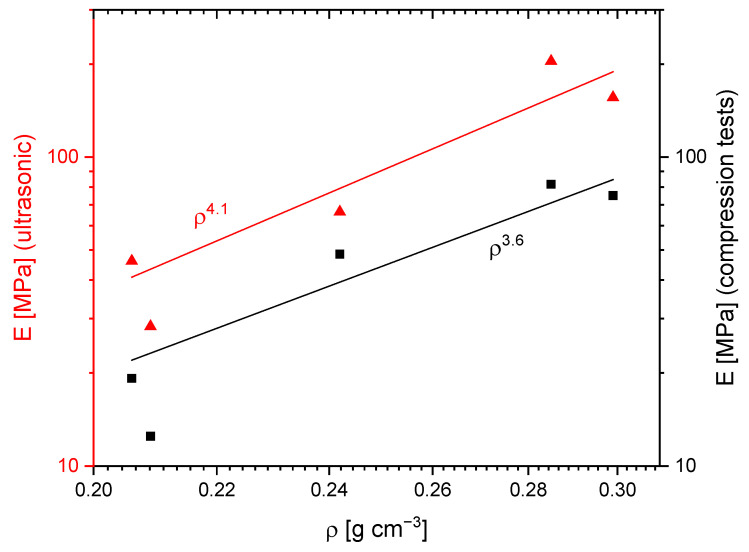
E as a function of density with a power-law relationship. On the left side, the E was calculated from ultrasonic run-time measurements (red), while on the right side E stems from compression tests (black).

Figure 8 further highlights the relationship between density and E. Scherdel et al. and Wong et al. reported that for aerogels, E correlates with density following an exponent of 3.6 [8,28]. For the present samples, an exponent of 4.1 was obtained from ultrasonic run-time measurements, potentially indicating that structural differences at the mesoscale, in addition to density, significantly influence E. However, given the limited number of data points (*n* = 5), the fit error is substantial, and the result is not statistically significant.

In contrast, compression tests yielded an exponent of 3.6, consistent with the literature values [8,28]. Nonetheless, the error in this fit is also considerable due to the small dataset. Additional data points would be necessary to reduce statistical uncertainties and validate these observations. Future studies focusing on mesoscale structural differences arising from varying gelation temperatures could provide further insights into their influence on the mechanical properties of silica gels.

## 3. Conclusions

This study has demonstrated the profound impact of gelation temperature on the structural, thermal, and mechanical properties of silica gels. By systematically varying gelation temperatures from 20 to 60 °C, we gained critical insights into how phase separation kinetics determine the resulting pore architecture, which, in turn, influences the functional performance of these materials. At lower gelation temperatures, slower kinetics promote more extensive phase separation, resulting in a bimodal and hierarchical pore structure characterized by large macropores and thicker mesoporous struts. This structure supports higher density and mechanical strength, as well as moderate thermal conductivity, due to the solid-phase connectivity and relatively large pores that facilitate gas-phase heat transfer.

As gelation temperature increases, faster phase separation limits the development of macropores, leading to a progressively more mesoporous structure with thinner struts and reduced mechanical stability. By 40 °C, the gels begin to lose their bimodal pore structure entirely and become increasingly mesoporous. Nitrogen sorption and MIP results reveal an increase in mesopore volume and mesoporosity proportion, reflecting the disappearance of macropores. SEM imaging corroborates this transition, showing a more homogeneously textured, finer pore network at higher gelation temperatures.

With the decrease in density, the solid-phase thermal conductivity decreases, reducing total conductivity. Additionally, the increase in mesoporosity and decrease in macropore size directly affect total thermal conductivity. As macropores diminish, gaseous thermal conductivity is increasingly limited by the Knudsen effect, where molecule–wall collisions dominate due to the small pore sizes, resulting in lower effective thermal conductivity. Thus, the combined effects of enhanced mesoporosity, Knudsen effects, and decreased solid connectivity contribute to the observed decline in thermal conductivity at higher gelation temperatures. This makes high-gelation-temperature gels particularly suitable for thermal insulation applications, where low thermal conductivity is crucial. Beyond thermal insulation, the sol–gel process offers exceptional flexibility in tailoring pore structures, making it a powerful approach for a wide range of applications. The ability to precisely control phase separation and gelation conditions allows for the design of materials with tailored structural characteristics, making them highly suitable for applications such as chromatography or catalysis. Notably, this study confirms that a redistribution of pore volume via the transition from a bimodal pore structure to predominantly mesoporous systems can be achieved even in large monoliths (2 × 2 × 6 cm^3^) without compromising structural integrity.

## 4. Materials and Methods

### 4.1. Chemicals

Tetraethyl orthosilicate (TEOS) and tartaric acid (TA) were procured from Sigma-Aldrich (St. Louis, MO, USA). Ethanol (100 vol.%) was obtained from VWR (Darmstadt, Germany), and PEO was supplied by Thermo Scientific (Waltham, MA, USA). All reagents were used as received, without further purification.

### 4.2. Synthesis

Initially, deionized water, PEO (molecular weight 100,000 g∙mol^−1^), ethanol, and TA were stirred until a homogeneous solution was obtained. TEOS was then added to the solution while maintaining continuous stirring. The molar ratios of the components were as follows: H_2_O:11.770, TEOS:1, PEO:0.00045, EtOH:0.765, and TA:0.224. Once hydrolysis commenced, the solution was poured into a polytetrafluoroethylene (PTFE) mold. The mold was placed in an autoclave and heated in an oven to induce gelation at specific temperatures (20, 30, 40, 50, or 60 °C). Throughout this study, the samples are designated as G_20, G_30, G_40, G_50, and G_60, where the number corresponds to the gelation temperature in degrees Celsius. Following a gelation time of 24 h, the gel was aged at 120 °C for an additional 24 h. Subsequently, the aged gel was immersed in an ethanol bath for solvent exchange, which was repeated twice.

#### 4.2.1. Supercritical Drying

Supercritical drying (SCD) was executed with CO_2_ in a custom-made autoclave (SEPAREX). The autoclave was run in a continuous process using a recycling line and release of the pore liquid (ethanol) with a separator. The SCD was performed at 60 °C and 120 bar.

#### 4.2.2. Calcination

To remove the polymer from the samples, a stepwise calcination process was applied. The samples were placed separately in a tube furnace and processed in a 5% oxygen and 95% nitrogen atmosphere with a total gas flow of 400 mL/min. The samples were first heated to 90 °C with a holding time of 5 h, followed by stages at 120 °C (5 h), 125 °C (5 h), and 150 °C (5 h), and finally heated to 300 °C with a holding time of 2 h. The heating rate was set to 0.5 K/min to ensure gradual decomposition and minimize thermal stress on the samples.

### 4.3. Characterization

#### 4.3.1. Density

The bulk density of the silica monoliths was determined based on their bulk volume from mass and geometrical measurements. Porosity and total pore volume were calculated based on the relationship between porosity and density, assuming a skeletal density of ρ_SiO2_ = 2.1 g∙cm^−3^ for the non-porous volume fraction. Porosity is defined as the total void fraction calculated from bulk and skeletal density.

#### 4.3.2. Nitrogen Adsorption

The N_2_-adsorption experiments were performed with a volumetric adsorption analyzer (ASAP 2020, Micromeritics, Norcross, GA, USA) at liquid nitrogen temperature (−196 °C). The mesopore volume was calculated extrapolating the isotherm to *p*/*p*_0_ = 1 and applying Gurvich’s rule.

#### 4.3.3. Mercury Intrusion Porosimetry

MIP was performed to analyze the meso- and macropore structures of the samples. Approximately 50 mg of each sample was placed in a dilatometer and evacuated to 0.2 mbar. The measurements were conducted using a Pascal 140 and Pascal 440 (Porotec, Hofheim, Germany), with mercury introduced into the samples at pressures up to 400 MPa. The pore diameter was calculated using the Washburn equation, assuming a mercury surface tension of 0.48 N·m^−1^ and a contact angle of 140°. To ensure that only macropores were analyzed, we exclusively considered pores larger than 50 nm.

#### 4.3.4. Scanning Electron Microscope

SEM images were acquired using a Zeiss ULTRA plus (Oberkochen, Germany). Prior to SEM imaging, the samples were sputter-coated with gold–palladium.

#### 4.3.5. Young’s Modulus

Ultrasonic run-time measurements with the pulse-echo technique were applied to determine the longitudinal sound velocity, and from that the elastic constant c11 [22,29]. The latter was converted to E by assuming a Poisson ratio of 0.2 [27].

#### 4.3.6. Compression Tests

The stress–strain behavior was tested by a double determination on cube-shaped specimens with an edge length of 10 mm. A universal testing machine ProLine Z010 (Zwick-Roell GmbH & Co. KG, Ulm, Germany) for a force range up to a maximum of 10 KN was used for the compression tests. In the test, a preload of 5 N was first applied to compensate for any inequality of the loaded surface. The test specimens were then exposed to displacement-controlled loading at a rate of 1 mm/min until failure. Young’s moduli were calculated using the linear regression functions for the roughly linear range in the stress–strain curve occurring immediately before the maximum force was reached.

#### 4.3.7. Thermal Conductivity

The thermal conductivity measurements were carried out using a self-made hot-wire set-up with automated gas pressure control at room temperature in nitrogen atmosphere with pressures ranging from 10^−1^ up to 10^5^ hPa [24]. To evacuate the samples, the silica gels were degassed for 1 h at 70 °C under vacuum prior to the measurement. Preliminary tests confirmed that this temperature was sufficient to achieve a stable vacuum without the need for higher temperatures. The pore size determination from thermal conductivity measurements was performed based on the Kaganer equation [24].

## Figures and Tables

**Table 1:** Effect of gelation temperature on the density, mesopore volume, and share of mesopore volume in the total pore volume. The mesopore volume was determined from nitrogen sorption.

Gelation Temperature °C	Density g·cm^−3^	Mesopore Volume ^1^ cm^3^·g^−1^	Total Pore Volume ^2^ cm^3^·g^−1^	Share of the Mesopore Volume in Total Pore Volume %
20	0.285	0.64	3.05	21
30	0.299	0.76	2.92	26
40	0.242	2.27	3.66	62
50	0.206	3.39	4.35	78
60	0.209	3.71	4.31	86

^1^ Mesopore volume determined from nitrogen sorption measurements. ^2^ Total pore volume calculated based on the relationship between bulk density and skeletal density.

**Table 2:** Effect of gelation temperature on macropore diameter, λ_1000hPa_, λ_evac_, and λ_gas,1000hPa_.

Gelation Temperature °C	Macropore Diameter ^1^ nm	λ_1000hPa_ mW (m∙K)^−1^	λ_evac_ mW (m∙K)^−1^	λ_gas,1000hPa_ mW (m∙K)^−1^
20	1212	68	38	30
30	632	62	34	28
40	157	38	22	26
50	106	32	20	12
60	71	27	19	8

^1^ Macropore diameters were determined using gas pressure-dependent thermal conductivity measurements.

**Table 3:** E and density of the samples.

Material	Density g∙cm^−3^	E (Ultrasonic) MPa	E (Compression Tests) MPa
G_20	0.285	204	82
G_30	0.299	156	75
G_40	0.242	67	48
G_50	0.206	46	19
G_60	0.209	28	12

## Data Availability

The original contributions presented in this study are included in the article; further inquiries can be directed to the corresponding authors.

## Supplementary Materials

The following supporting information can be downloaded at: https://www.mdpi.com/article/10.3390/gels11030196/s1, Figure S1: SEM images showing the mesoporous structure of the individual samples at 100,000× magnification.
