# Supplementary material for: Influence of Gelation Temperature on Structural, Thermal, and Mechanical Properties of Monolithic Silica Gels with Mono- and Bimodal Pore Structure
Source: Gels. 2025 Mar 12;11(3):196. doi: 10.3390/gels11030196 (PMC11942077; doi:10.3390/gels11030196)
Supplement: Supplementary file 1 [file gels-11-00196-s001.zip › gels-3505059-supplementary.pdf]

Supplementary Materials

Influence of Gelation Temperature on the Structural, Thermal, and Mechanical Properties of Monolithic Silica Gels with Mono- and Bimodal Pore Structure

Kai Müller [1,], Christian Scherdel [2], Stephan Vidi [2], Gudrun Reichenauer [2], Moritz Boxheimer [3], Frank Dehn [3] and Dirk Enke [1]*

[1] Institute of Chemical Technology, Leipzig University, 04103 Leipzig, Deutschland; dirk.enke@uni-leipzig.de
[2] Center for Applied Energy Research, 97074 Würzburg; Christian.scherdel@cae-zerocarbon.de
[3] Institute of Concrete Structures and Building Materials (IMB), Karlsruhe Institute of Technology, 76131 Karlsruhe, Germany; frank.dehn@kit.edu
* Correspondence: kai.mueller@uni-leipzig.de

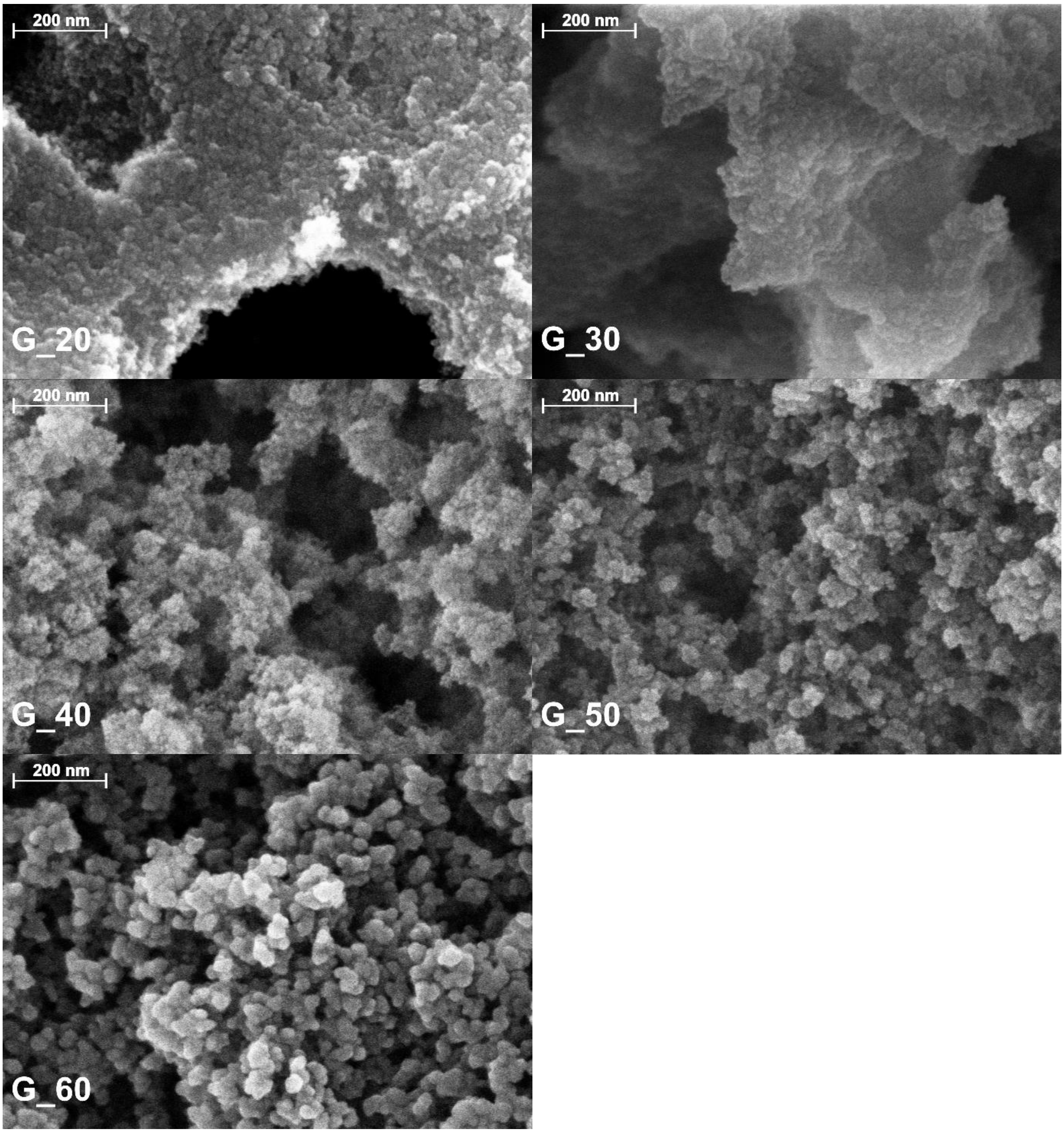

Figure S1. SEM images showing the mesoporous structure of the individual samples at 100,000x magnification.
